# Specific Graft Treatment Solution Enhances Vascular Endothelial Function

**DOI:** 10.31083/j.rcm2311368

**Published:** 2022-10-28

**Authors:** Attila Kiss, Petra Lujza Szabo, Christopher Dostal, Zsuzsanna Arnold, Daniela Geisler, Ingo Crailsheim, Sandra Folkmann, Martin Grabenwöger, Bruno Karl Podesser, Bernhard Winkler

**Affiliations:** ^1^Ludwig Boltzmann Institute for Cardiovascular Research at the Center for Biomedical Research, Medical University Vienna, 1090 Vienna, Austria; ^2^Department of Cardio-Vascular Surgery Vienna Heart Center Clinic Floridsdorf and Karl Landsteiner Institute for Cardio-Vascular Research, 1210 Vienna, Austria

**Keywords:** vein graft, preservation solution, endothelium, coronary artery bypass grafting, myograph

## Abstract

**Background:**

Saline is still the most widely used storage and rinsing solution for vessel grafts during cardiac surgery despite knowing evidence of its negative influence on the human endothelial cell function. Aim of this study was to assess the effect of DuraGraft©, an intraoperative graft treatment solution, on human saphenous vein segments and further elaborate the vasoprotective effect on rat aortic segments in comparison to saline.

**Methods:**

Human Saphenous vein (HSV) graft segments from patients undergoing aortocoronary bypass surgery (n = 15), were randomized to DuraGraft© (n = 15) or saline (n = 15) solution before intraoperative storage. Each segment was divided into two subsegmental parts for evaluation. These segments as well as rat aortic segments stored in DuraGraft© underwent assessment of vascular function in a multichamber isometric myograph system in comparison to Krebs-Henseleit solution (KHS), a physiologic organ buffer solution.

**Results:**

Potassium-Chloride (KCL)-induced contraction depicted a tendency towards increase when treated with DuraGraft© compared to saline preservation of HSV segments (23.02 ± 14.77 vs 14.44 ± 9.13 mN, *p* = 0.0571). Vein segments preserved with DuraGraft© showed a significant improvement of endothelium-dependent vasorelaxation in response to cumulative concentrations of bradykinin compared to saline treated segments (*p *< 0.05). Rat aortic segments stored in saline showed significantly impaired vasoconstriction (3.59 ± 4.20, *p *< 0.0001) and vasorelaxation when compared to KHS and DuraGraft© (*p *< 0.0001).

**Conclusions:**

DuraGraft© demonstrated a favorable effect on graft relaxation and contraction indicating preservation of vascular endothelial function.

**Clinical Trial Registration Number:**

NCT04614077.

## 1. Introduction

Coronary artery bypass grafting (CABG) continues to be the “gold standard” for patients with complex multivessel coronary artery disease because of the superior long-term outcome. In the modern era of arterial revascularization, saphenous vein grafts (SVGs) still remain the most often used conduits for CABG worldwide [1, 2]. As widely known the graft patency is influenced by multiple factors. One of these is intimal hyperplasia progressing to vein-graft disease and graft failure [3]. Various aspects and procedures have been studied and published with fundamental improvements like pedicled harvesting technique, no touch methods and of course external stenting of the vein graft [4, 5]. However, one topic was neglected for a long period but currently gains more and more attention [6]. The type of graft storage, flushing and rinsing solution that is intraoperatively used to store the conduits can largely influence endothelial integrity and vessel function [7]. Since the moment of grafting is the last time when the surgeon can influence the “auto-transplanted” vessel, every measure and care should be taken to ensure the best possible long-term outcome. This includes the intraoperative storage and flushing solution. The benefits of meticulous surgical handling, training, experience and various precautions during harvesting are redundant if the procedure is interfered by using the incorrect storage solution. In line with that, a subsection of the PREVENT IV trial demonstrated that using a buffered solution resulted in lower vein graft failure rates when studied by angiography at 12 to 18 months after surgery and showed generally a better clinical outcome compared to non-buffered acidic saline (pH value of 5.5) or autologous whole blood solutions (AWB) [8]. Since AWB is only beneficial as long as inside the intact circulation and harmful once outside e.g. in the operative setting, due to an alkalytic pH (above 8) as a result of ${\mathrm{CO}}_{2}$ loss we did not include AWB in the current experiments. The results for AWB in the literature are well reported and are undoubtedly not beneficial for the endothelium [9, 10]. Still heparinzed saline and even AWB are in use in the clinical setting, not only in coronary procedures but also for peripheral vascular surgery where the same principles apply. Therefore, novel approaches should limit the early endothelial dysfunction or preserve endothelial integrity to boost graft patency which has become a highly demanded clinical goal these days.

The current study compared the impact of intraoperative preservation of SVGs from patients undergoing CABG in a specific ionically balanced storage solution (DuraGraft®, Maryzyme Inc, Jupiter, FL, USA) versus saline solution in the isolated organ bath testing system. DuraGraft®, is also a pH-balanced physiological salt solution containing L-glutathione, L-ascorbic acid, L-arginine and other additives that protect the graft from the damaging effects of ischemia and handling during CABG. DuraGraft® is a CE marked intra-operative graft storage solution and currently approved in Europe and several other global health care systems.

Since this is currently the only specific storage solution DuraGraft® was the target solution in this study to be compared to the still most widely used solution saline. In addition further characterization of the impact of saline on vascular endothelial function in rat aortic segments and the direct influence on human umbilical vein endothelial cells (HUVECs) was undertaken to specify initial findings.

## 2. Patients and Methods

The study was approved by the local Ethics Committee Nr. EK-20-219-1020 of the City of Vienna/Austria and registered as observational study by ClinicalTrials.gov. under the number NCT04614077.

Saphenous vein segment remnants were collected from 15 CABG patients after their informed consent. Patient’s details are presented in Table 1. Within 15 patients undergoing aortocoronary bypass surgery, saphenous vein graft segments were randomized to DuraGraft© (n = 15) or heparinized saline (B Braun AG, Melsungen, Germany) (n = 15) solution before intraoperative storage. Each 2 cm long segment of human saphenous vein (HSV) was divided into two 1 cm long parts for twofold evaluation. In total n = 28 HSV segments were collected and n = 23 segments/conditions were used for endothelial-dependent and endothelial-independent vasorelaxation assessment. Special care was taken to exclude patients with concomitant diseases or medical treatment that could interfere with the outcome, testing methods with special focus on vessel wall reactivity and further pathophysiological vascular conditions.

**Table 1.** **Descriptive patients characteristics preoperative**.

Patients characteristics and cardiovascular risk factors	
patients, n/ (female %)	15/ (18%)
Age, median (years)	66.9
Body mass index (BMI), median	27.2
Hypertension, n (%)	10 (67%)
Dyslipidemia, n (%)	15 (100%)
Diabetes mellitus (any), n (%)	7 (46.7%)
Smoker active, n (%)	7( 46.7%)
Chronic renal insufficiency, n (%)	4 (26.7%)
Dialysis, n (%)	4 (26.7%)
Pulmonary hypertension, n (%)	3 (20%)
Chronic obstructive pulmonary disease, n (%)	4 (26.7%)
Atrial fibrillation, n (%)	3 (20%)
Peripheral artery disease, n (%)	4 (26.7%)
Central artery disease, n (%)	3 (20%)
Prior percutaneous intervention, n (%)	4 (26.7%)
Previous myocardial infarction, n (%)	6 (40%)
Heart valve disease, n (%)	2 (13%)
Preoperative medication	
Nitrates, n (%)	0 (0%)
Calcium blockers, n (%)	3 (20%)
Beta blockers, n (%)	10 (67%)
Renin-angiotensin system inhibitors, n (%)	2 (13%)
Diuretics, n (%)	4 (26.7%)
Aspirin	15 (100%)
Statins	15 (100%)

n, numbers of patients; % indicates percentage.

The following inclusion criteria were applied:

Age between 18–80 years.

Planned CABG operation.

Suitable vein grafts with the absence of blow outs, varicous veins or previous stripping.

Exclusion criteria:

Age <18 or >80 years.

Emergency CABG.

Preoperative myocardial infacrtion <48 h.

Re- operation.

Prior PCI <48 h.

Ejection fraction (EF) <30%.

Severe organ dysfunction (any malignancy, sepsis <5 days, life expectancy <3 years).

Pregnant women were not included.

Any disease of the lower veins.

Any vasculitis.

Hemoglobin A1c (HbA1c) levels >6.5% (mmol/mol).

The segments of human saphenous vein were harvested in open technique. All patients underwent preoperative ultrasound scanning of the vein segments and varicose veins (outer diameter above 3.5 mm) were excluded. Special care was taken not to stretch by brisk handling or to touch the vessel frankly during the harvest procedure, therefore vein grafts were only harvested by experienced surgeons.

### 2.1 Assessing Vasoactivity in Human Saphenous Vein Segments Using Wire Myograph

Human Saphenous vein segments were cut out in 20 mm pieces, carefully pressure controlled flushed with 10 mL (mL, NaCl or DuraGraft®) at room temperature and placed in the solution they were assigned to (NaCl or Duragraft), any contact with any other substance was fully avoided. There was no mixture of the substances. Flushing, storage and testing was undertaken only with the given solution (NaCl or DuraGraft®). Each 20 mm long vein segment was divided into two study samples accounting for a total of 28 samples, as two sub-segments had to be excluded. The segments were then put immediately into pre-oxygenated (45 minutes oxygenation time) DuraGraft© or saline solution at normal room temperature and transferred to the laboratory in a sterile isolated box. The segments were kept under these conditions for a total of 60 minutes and then put into cold and oxygenated (5% ${\mathrm{CO}}_{2}$ and 95% $O_{2}$) Krebs-Henseleit solution (KHS) containing (in mM/L) 119 NaCl, 4.7 KCl, 2.5 ${\mathrm{CaCl}}_{2}$•2 $H_{2}$O, 1.17 ${\mathrm{MgSO}}_{4}$•7 $H_{2}$O, 20 ${\mathrm{NaHCO}}_{3}$, 1.18 ${\mathrm{KH}}_{2}$${\mathrm{PO}}_{4}$, 0.027 EDTA, 10.5 glucose) and the segments were gently cleaned from all connective tissue using Zeiss stereo preparation microscope (Carl Zeiss Meditec AG, Jena, Germany). After cleaning the parts of the saphenous vein, the subsegments were cut into 2 mm pieces and mounted onto a multi-chamber isometric myograph system (Model 620M, Danish Myo Technology, Aarhus, Denmark). The single organ chambers of the myograph were filled with heated (37 °C) and oxygenated KHS and each individual chamber was further heated and bubbled with oxygen during the whole procedure. To determine the resting tension we used the AD Instruments’ LabChart® DMT Normalization Module (ADInstruments Inc., Colorado Springs, CO, USA) to mimic physiological conditions (target pressure: 20 mmHg). Segments were allowed to equilibrate for 30 minutes and resting tension was continuously adjusted during this period as described previously [11]. Reference contractions were elicited by hyperkaliaemic (124 mM, KCl) solution. Precontraction of the human saphenous vein was achieved by norepinephrine (NE, 1 μM, Arterenol, Sanofi), respectively. Endothelial dependent and independent relaxation was tested by the cumulative dosage of bradykinin (Bra, 1 nM–10 μM, a nitric oxide-dependent vasodilator, Sigma Aldrich) and sodium nitroprusside (SNP, 0.1 nM–1 μM, a nitric oxide-independent vasodilator Merck), respectively. The data were continuously recorded using the software program LabChart Pro (ADInstruments Inc., Colorado Springs, CO, USA).

### 2.2 Assessing Vasoactivity in Rat Aortic Segments Using Wire Myograph

To further test the potential vascular protective effects of DuraGraft©, segments of the abdominal aorta were used from Sprague Dawley rats. Male adult Sprague-Dawley rats (12–14 weeks old, body weight of 350–380 gram; Department for Laboratory Animal Science and Genetics, Himberg, Austria) were used. The experimental protocol was approved by the Ethics Committee for Laboratory Animal Experiments at the Medical University of Vienna and the Austrian Ministry of Science and Research (BMWF-66.009/0023-WF/V/3b/2016) and conforms with the Guide for the Care and Use of Laboratory Animals, published by the US National Institutes of Health (NIH Publication No. 85-23, revised 1996) [12, 13]. Briefly, rats were anaesthetized by intraperitoneal injection of a mixture of Xylazine (4 mg/kg; Bayer, Leverksen, Germany) and Ketamine (100 mg/kg; Dr E. Gräub AG, Switzerland), heparin was injected (iv. femoral artery) and the abdominal aorta was collected as described previously [12].

After cleaning, the segment was cut into 2 mm sections and mounted onto a multi-chamber isometric myograph system (Model 620M, Danish Myo Technology, Aarhus, Denmark). The chambers were filled with one the following solution: (1) KHS (gold standard, positive control), (2) DuraGraft© and (3) physiological saline (0.9%). Each individual chamber was further heated and bubbled during the whole procedure. To determine the resting tension the AD Instruments’ LabChart® DMT Normalization Module to mimic physiological conditions was used with a target pressure of 100 mmHg. Segments were allowed to equilibrate for 45 minutes and resting tension was continuously adjusted during this period as described previously [11, 14]. Reference contractions were elicited by hyperkaliaemic (124 mM, KCl) solution. Precontraction of the aorta segments was achieved by Phenylephrine (PE, 1 nM–1 μM, Sigma Aldrich). Endothelial dependent and independent relaxation was tested by the cumulative dosage of Acetylcholine (ACh, 1 nM–10 μM, a nitric oxide-dependent vasodilator, Sigma Aldrich) and sodium nitroprusside (SNP, 0.1 nM–1 μM, a nitric oxide-independent vasodilator Merck), respectively.

### 2.3 Human Umbilical Vein Endothelial Cells (HUVEC) Cultivation and Cell Viability Measurement

Human umbilical vein endothelial cells (HUVECs, Lonza, Basel, Switzerland) were cultured in Medium 200 supplemented with Large Vessel Endothelial Supplement (LVES), 10% foetal bovine serum (FBS) and 1% penicillin and streptomycin solution and maintained at 37 °C and 5% ${\mathrm{CO}}_{2}$. The cells were cultured on 96 well plates at least for 24 hours. HUVECs were treated either with 100 μL of (1) Medium 200 served as control, (2) KHS, (3) DuraGraft© solution or (4) saline solution for 30 and 60 minutes followed by cell viability measurement. Briefly, XTT sodium salt was dissolved in warm PBS (1 mg/mL, Santa Cruz Biotechnology) and phenazine methosulfate was added in 25 μM final concentration. 50 μL of XTT solution was used and the cells were incubated for 3–4 hours for the colour reaction. Absorbance was measured at 450/630 nm wavelength with a plate reader (Tecan SparkControl Magellan V2.2, Männedorf, Switzerland).

### 2.4 Materials and Reagents

All chemicals were purchased from Sigma Aldrich (Sigma Inc. Burlington, MA, USA) unless otherwise specified. Preservation solutions for all experiments contained 10 units/mL unfractionated Heparin. DuraGraft© was purchased from Somahlution Inc, Jupiter, Florida, United States.

### 2.5 Data Analysis

The contractile response was defined by the stress, which was calculated using the force generated by the vein rings. Vascular relaxation to Bradykinin and ACh was expressed as percentage of contraction to NE or PE, respectively. Differences in concentration-dependent relaxations induced by Bradykinine and ACh were analysed using two-way ANOVA followed by Bonferroni’s test when appropriate. Differences between multiple groups were analysed using one-way ANOVA followed by Bonferroni’s test. The number of experimental observations (n) refers to the number of vascular segments in respective experiments.

To demonstrate the good comparability of all cohorts, statistical testing for differences in baseline, procedural, and follow-up data has been performed. Depending on the variable’s distribution continuous data are either expressed as means and standard deviation (+/- SD) or median and were analyzed with one-way analysis of variance (ANOVA). Categorical variables are expressed in absolute numbers and percentages.

All data were expressed as mean ± SD and were calculated using GraphPad Prism (Version 7.03; GraphPad Software Inc., San Diego, CA, USA).

Statistical significance was accepted when *p *< 0.05.

## 3. Results

We took maintenance of pH was vital to physiologic function and cellular viability of SVGs as given. In addition, it is important to state therefore that SVG segments after 30–60 minutes incubation with saline or DuraGraft© were transferred into Krebs solution and subsequently all measurements were performed in Krebs solution in this study.

###  3.1 Effect of Preservation Solution on Contractile Responses in SVGs

**Fig. 1.**
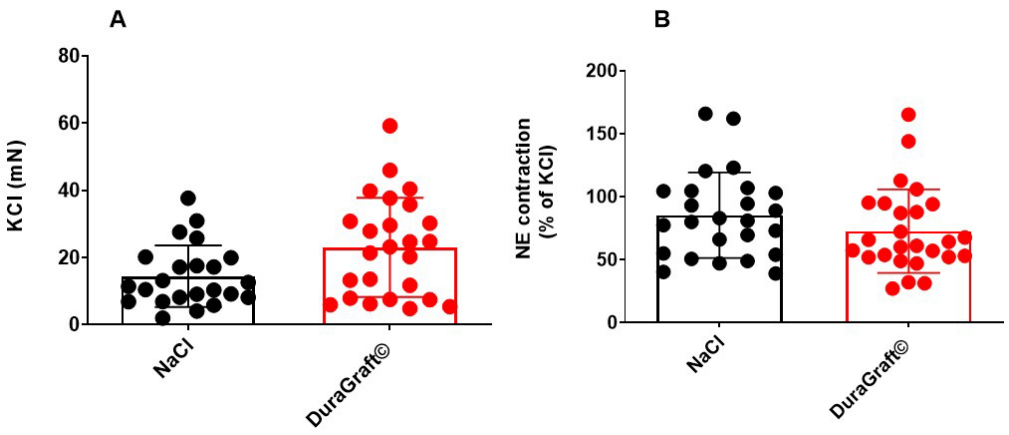
**Effect of preservation solution on contractile responses in HSV**. (A) In response to the high $K^{+}$ (KCl) Krebs solution, the HSV segments showed tendency toward increase when stored in DuraGraft© compared to normal saline preservation (23.02 ± 14.77 vs 14.44 ± 9.13 mN, *p* = 0.0571). (B) In response to NE, HSV segments showed no difference between the storage conditions (Saline 85.31 ± 33.9% and DuraGraft© 72.66 ± 33.13%; *p* = 0.167). The NE response is expressed as percentage of KCl contraction. n = 15 patients and n = 23–25 segments/condition.

Maximal contraction in response to KCl (124 mmol) in Krebs solution, showed a tendency towards increase in contraction in DuraGraft© when compared to normal saline preservation HSV (23.02 ± 14.77 vs 14.44 ± 9.13 mN, *p* = 0.0571; segments/group, Fig. 1A). Next, when the resting tension reached a stable baseline, no attempt was made to adjust the HSV tension. After the equilibrium, the HSV segments were primed by the addition of norepinephrine (1 × ${\mathrm{10}}^{-6}$ M) to the organ bath in order to measure the maximal contraction in response to α_1_ adrenoceptor activation. There was no difference in maximal contraction (% of KCl contraction) achieved by NE between the groups (Saline 85.31 ± 33.9% and DuraGraft© 72.66 ± 33.13%; *p* = 0.167, Fig. 1B).

### 3.2 Effect of Preservation Solution on Endothelial-Dependent and Independent Vasorelaxation in HSV

**Fig. 2.**
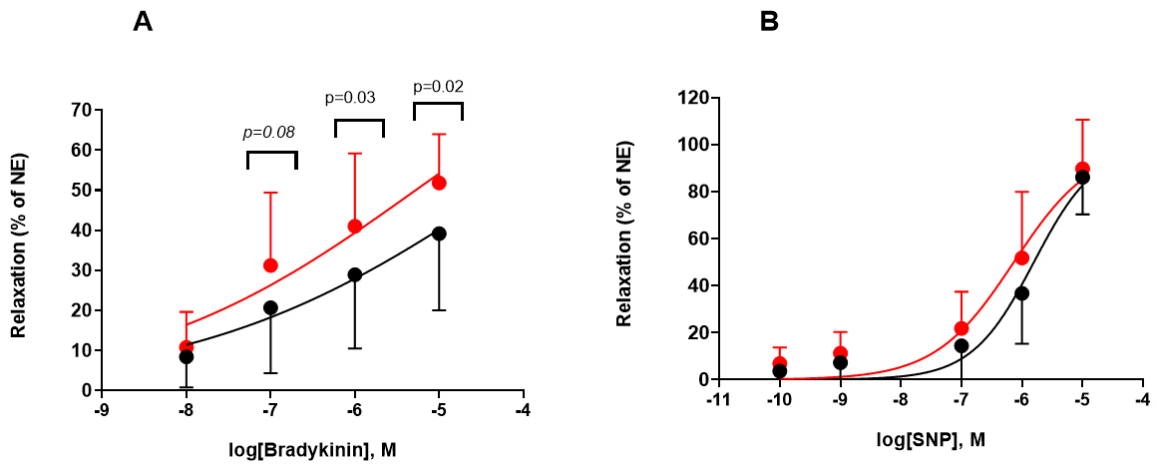
**Endothelial dependent and independent vasorelaxation-saphenous vein grafts**. Effects of NaCl (black) and DuraGraft® (red) on vascular reactivity in the saphenous vein grafts. (A) Vein rings were precontracted with NE and relaxed with the cumulative dosages of Bradykinin. The Bradykinin response is expressed as percentage of the maximum NE response and baseline tension. (B) Sapneouse vein were precontracted with NE and relaxed with the cumulative dosages of sodium nitroprusside (SNP). The SNP response is expressed as percentage of the maximum NE response and baseline tension. Data are expressed as mean ± SD, n = 15 patients n = 23 segments/condition.

To investigate the potential protective efficacy of DuraGraft© on the vascular endothelium, we assessed the vascular reactivity of HSV segments in patients planned for elective CABG. The HSV segments were stored in DuraGraft© showed a significantly preserved endothelium-dependent vasorelaxation in response to cumulative dosage of Bradykinin in comparison to saline stored segments (Fig. 2A, *p *< 0.05). Endothelium-independent vasorelaxation was assessed by the response to cumulative dosage of SNP, and there was no difference between the two storage conditions (Fig. 2B).

### 3.3 Vasoconstriction and Endothelial-Dependent Relaxation in Rat Aortae

**Fig. 3.**
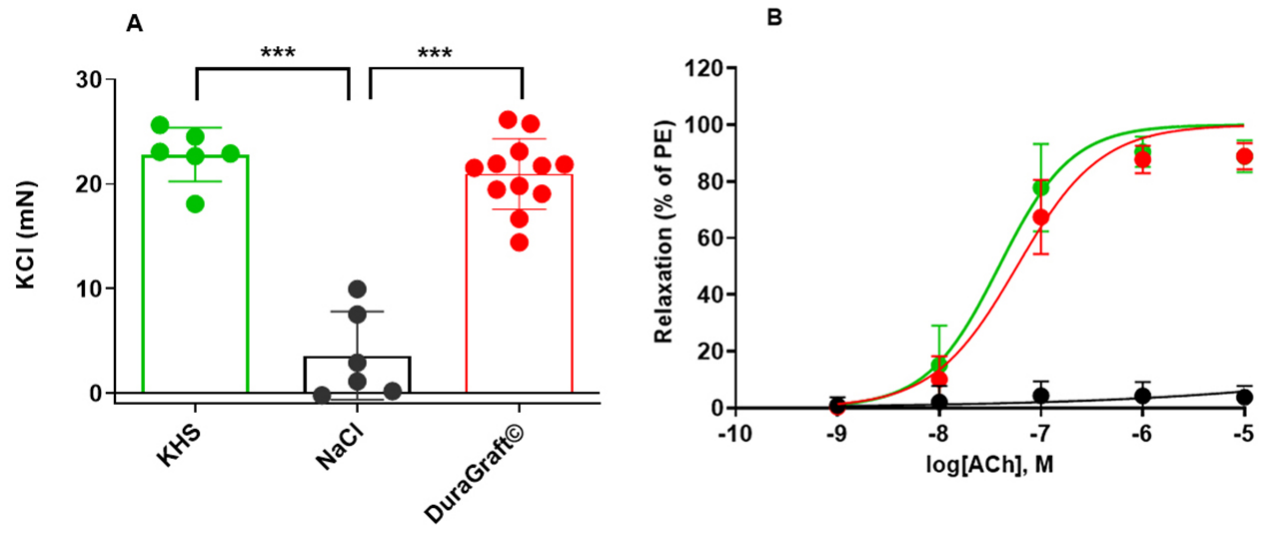
**Contractile response and endothelial dependent vasorelaxation-rat aorta**. (A) Aorta segments that were kept in DuraGraft© or Krebs-Henseleit solution (KHS) showed comparable response to KCl (contraction; 20.97 ± 3.37 mN vs 22.87 ± 2.57 mN) until saline stored segments showed significant impairment in vasoconstriction (3.59 ± 4.20 mN, *p *< 0.0001). (B) Rat aortic rings were precontracted with PE and relaxed with the cumulative dosage of ACh Physiological treated aorta segments had a significantly impaired endothelial function compared to both DuraGraft© or KHS treated ones (***p < 0.0001 KHS vs saline; ^###^*p *< 0.0001 DuraGraft© vs saline). Data are mean ± SD, n = 3 rats and 4 segments/rat.

In the next step, rat aorta segments were used as a model of normal vascular tissue to further characterize and compare the vascular protective effects of DuraGraft© solution. The myograph chambers were filled with KHS, saline and DuraGraft© and the aorta segments from rat were mounted. Aorta segments that were kept in DuraGraft© showed comparable response to KCl (contraction; 20.97 ± 3.37 vs 22.87 ± 2.57 mN, Fig. 3A) and ACh (endothelium dependent relaxation; Fig. 3B) as KHS solution. In contrast, the aorta segments were kept in physiological saline showed significant impairment in response to both vasoconstriction (3.59 ± 4.20, *p *< 0.0001, Fig. 3A) and vasorelaxation when compared to Krebs Solution as well as DuraGraft© preservation (Fig. 3B, *p *< 0.0001), respectively.

### 3.4 Cell Viability in HUVECs

**Fig. 4.**
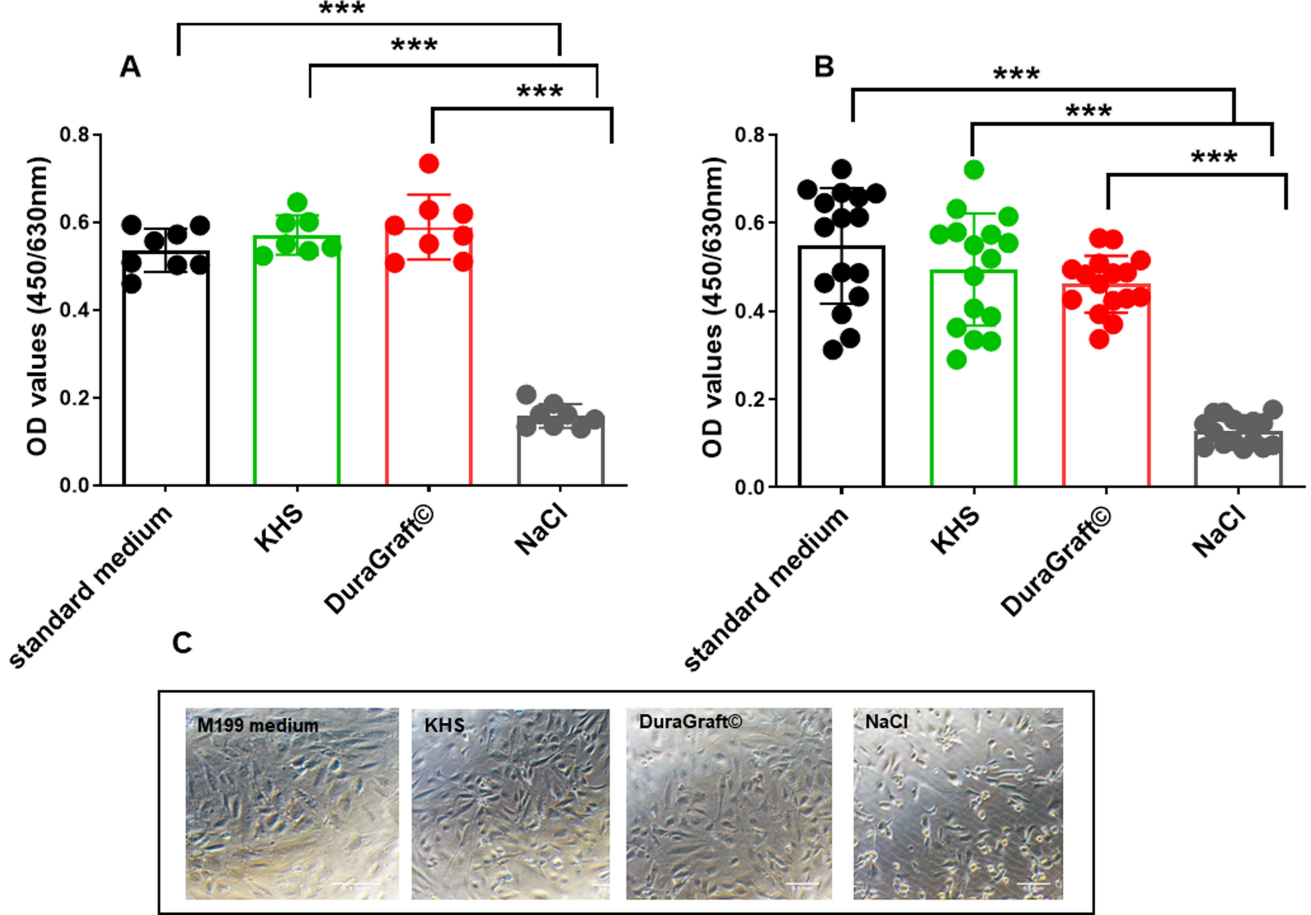
**Human Umbilical Vein Endothelial Cells-Viability**. Effects of standard medium, NaCl, DuraGraft® and Krebs-Henseleit solution (KHS) on HUVECs viability after (A) 30 minutes and (B) 1 h incubation with the respective conditions. (C) Representative images of the cell morphology under the respective conditions after 1 h. Data are mean ± SD, n = 7–16 replicates/condition ***p < 0.0001 vs saline (NaCl).

Fig. 4C displays the morphology of HUVECs cultured in control medium (M200 Medium). The cells were aliquoted and kept in one of the following conditions: (1) control group (M200 Medium), (2) KHS, (3) physiological saline and (4) DuraGraft© solution for 30 minutes or 60 minutes. Then the cell viability was evaluated by XTT assay. Saline treatment for 30 or 60 minutes markedly inhibited cell viability (Fig. 4A,B compared to control, KHS and DuraGraft©, respectively (*p *< 0.0001). Of importance, cell viability was similar between the control and DuraGraft© group after 30 or 60 minutes incubation (Fig. 4C), suggesting DuraGraft© maintains endothelial cells viability and metabolism, indicating the fact that it is an optimal solution for preserving endothelial cells viability and function for at least 60 minutes. In addition, HUVECs were cultured under M200 Medium, KHS or DuraGraft© presented a confluent elongated shape, while those that were subjected to saline became spherical and non-confluence.

## 4. Discussion

Previous studies have already suggested that preservation in physiologic saline may harm vascular conduits and can accelerate the development of neointimal hyperplasia formation [3, 5, 6, 7, 8]. Still saline is one of the most or the most widely used solution for intraoperative graft storage or graft flushing besides AWB preparations or individual mixtures. Saline if non buffered has an acidic pH value of 5.5, the physiological pH of circulating blood is 7.32 pH. AWB becomes alkalotic once outside the humas circulation as described above. As Veres *et al*. [15] stated in 2015 storage with physiological saline and heparinized blood solutions is unable to protect the endothelium against cold ischaemia and warm reperfusion injury. Already in 2014, Harskamp *et al*. [8] examined the influence of the preservation solutions on vein graft failure using data from the PREVENT IV trial. Grafts were randomized to different groups of preservation solution consisting of saline, buffered saline and autologous whole blood. Grafts stored in buffered saline had significantly lower one-year vein graft failure rates compared to the other two groups, and were associated with a lower risk of five-year death, myocardial infarction and secondary revascularization, suggesting that intraoperative graft preservation is one of the key procedures in order to reduce graft failure risk. Despite these important clinical findings, heparinized normal saline is still widely used in coronary artery bypass grafting. Interestingly the first representative study was by O’Connell *et al*. [16] in 1974, conducted on the intima of arterial and not venous grafts. This study clearly demonstrated negative effects of normal saline (NS) on vascular endothelium and graft patency in a rabbit model. The topic of a specific graft storage or even treatment solution was neglected in cardiovascular research but gains in recent years more and more interest [6, 7, 17]. The data from the current study showed a clear positive effect for DuraGraft© as a representative for a specific solution. As presented in the results the HSV segments that were preserved with DuraGraft© showed significantly preserved endothelium-dependent vasorelaxation in response to cumulative dosage of Bradykinin when compared to saline preservation. The solution itself is buffered and upholding the cell metabolism due to preserving glucose levels but also reducing oxidative stress and amino acid (L-Argine) related vasodilatation [17]. The product is a relatively novel solution against endothelial-damage developed to efficiently protect the structural and functional integrity of the vascular endothelium. DuraGraft© is described as structural and functional endothelial stabilizer in aortocoronary bypass surgery, antioxidative, radical-scavenging, nitric oxide (NO)-synthetize-supporting, anticoagulant, isotonic structural and functional endothelial stabilizer for graft stabilization during venous and arterial aortocoronary bypass surgery. Saline does of course not provide any metabolism upholding elements and if not buffered has a direct damaging acidic effect on the endothelium. DuraGraft© alleviated in a recent study vascular function *in vitro* following ischemia- reperfusion injury [18]. These results although representing data from *in vitro* animal studies were in line with the findings of this study conducted on human saphenous vein segements.

Currently a prospective observational registry with DuraGraft© targeting at 3000 patients undergoing an isolated CABG procedure or a combined procedure with at least one saphenous vein grafts or one free arterial graft is finished: EU Multicenter Registry to Assess Outcomes in CABG Patients: Treatment of Vascular Conduits With DuraGraft [VASC]. Data on baseline, clinical, and angiographic characteristics as well as procedural and clinical events were and will be collected [3, 17, 19]. Because preservation in the buffered solution represented by DuraGraft© appeared to be superior to non-buffered saline in isolated rat aorta segments and relaxation in HSV, we concluded that maintenance of pH could be vital to physiologic function and cellular viability of HSV. Furthermore, a recent study by Tekin *et al*. [20] demonstrated that SVG stored in DuraGraft© had lower oxidative level and higher antioxidant capacity, both may contribute and partially explain the preservation of endothelial function as observed in another study by Szabó *et al*. [12]. In addition, it is important to state that SVG segments after 40–60 min (comparative time as in the Operating room) incubation with saline or DuraGraft© were transferred and then kept in KHS solution with all measurements performed under this conditions, suggesting DuraGraft© storage solution effectively alleviates endothelial dysfunction [21].

As the next step further characterization to compare the vascular protective effects of DuraGraft© solution on rat aorta segments as a model system of healthy vascular tissue was conducted. The aorta segments that were kept in DuraGraft© showed comparable response to Potassium-Chloride and endothelium dependent relaxation solution. In contrast, the aorta segments that were stored in saline showed significantly impaired vasoconstriction and vasorelaxation when compared to KHS and DuraGraft© preservation. In line with the initial findings, DuraGraft® alleviates vascular dysfunction following ischemia and reperfusion injury by reducing nitro-oxidative stress and the expression of intercellular adhesion molecule-1 (ICAM-1), without leukocytes engagement in the rat model [12]. Furthermore, the study by Pachuk *et al*. [7] in 2019 displayed in pig mammary arteries results exactly in line with the data above for an animal model set up for healthy vessel segments. Loss of HSV graft-cell viability was observed as early as 15 minutes post-exposure to saline whereas viability was maintained up to 5 hours’ exposure to DuraGraft©. Histological analyses performed on pig mammarian artery (PMVs) demonstrated endothelial damage in PMVs stored in saline. Cytotoxicity assays demonstrated that saline-induced microscopically visible cell damage occurred within 60 minutes [22, 23]. In line with these findings, cell viability was evaluated in HUVECs by XTT assay in this study, saline (30 and 60 minutes) treatment markedly inhibited cell viability when compared to the control KHS and DuraGraft©, respectively (*p *< 0.0001). Of importance, cell viability was similar between the control and DuraGraft© group after 30 and 60 minutes incubation. These data suggested that DuraGraft© maintains endothelial cell viability and metabolism. DuraGraft© representing a specific storage solution the data of this study votes for the use of a specific solution for preserving endothelial cell viability and function in vascular procedures. Maintaining endothelail cell integrity may be also important to reduce the risk of graft occlusion, myocardial infarction and repeat-revascularisation due to early endothelial affection and later graft failure. In this context, recent clinical studies demonstrated that intraoperative graft treatment with DuraGraft showed a favorable effect on saphenous vein graft wall thickness compared to saline treatment at 12 months follow up period [12, 15, 18, 24, 25]. Current literature shows additionally that not only saphenous vein grafts but also arterial grafts benefit from a specific treatment solution. A recent study by Aschacher *et al*. [14] depicted lower levels of reactive oxygen species (ROS) after the treatment Interestingly in this study an increased expression of transforming growth factor β (TGFβ), platelet-derived growth factor α/β (PDGFα/β), and heme oxygenase-1 (HO-1) which are indicative for vascular protective function was reported after Duragraft exposure. Once more, as summarized in the literature and detected in this triple approach, saline is clearly not beneficial for the human endothelium whilst DuraGraft© being a representative for a specific storage and treatment solution demonstrated a significantly positive, at least superior to non-buffered saline solution effect. This study and the current results call for the stop of saline as vascular storage and graft flushing solution and for the use of a specific agent instead [7, 12, 14, 15, 17, 18, 19, 21, 22, 23, 24, 25, 26, 27].

## 5. Limitations

The study was limited by its single center design and patient’s numbers in terms of the HSV samples. Although specific care was taken to avoid any influence on the vessel and endothelium in terms of preparation or transport some undetectable risk factors or patient’s details might be present but not obvious at the time of hospitalization. In addition, animal study experiments confirm that DuraGraft© is as efficient as KHS in respect of vascular reactivity, and superior than saline. However, we have not stored the segments of aorta (rat) either in saline or that DuraGraft© prior to performing vascular reactivity assessment and performed on isolated aortic not on venous segments. However, we do not anticipate a difference between the artery and the venous segment in respect to vascular protection by Duragraft©. This study represented a momentum snapshot of the influence and further longterm data is urgently needed to confirm the protective effects. The author WB is participating in the European Multicenter Trail VASC as national PI. Nevertheless, the protective effect of DuraGraft© was demonstrated on human specimens in this study.

## 6. Conclusions

Saline is still the most widely used storage and flushing solution for vessel grafts during cardiac surgery. Saline is clearly not beneficial for the human endothelium whilst DuraGraft© being a representative for a specific storage and treatment solution demonstrated a positive effect. This study and the current results call for the stop of saline as vascular storage and graft flushing solution and for the use of a specific agent instead.

## References

[1] Wilbring M, Tugtekin SM, Zatschler B, Ebner A, Reichenspurner H, Kappert U (2013). Preservation of endothelial vascular function of saphenous vein grafts after long-time storage with a recently developed potassium-chloride and N-acetylhistidine enriched storage solution. *The Thoracic and Cardiovascular Surgeon*.

[2] Carrel T, Winkler B (2017). Current trends in selection of conduits for coronary artery bypass grafting. *General Thoracic and Cardiovascular Surgery*.

[3] Caliskan E, Sandner S, Misfeld M, Aramendi J, Salzberg SP, Choi YH (2019). A novel endothelial damage inhibitor for the treatment of vascular conduits in coronary artery bypass grafting: protocol and rationale for the European, multicentre, prospective, observational DuraGraft registry. *Journal of Cardiothoracic Surgery*.

[4] Ragnarsson S, Janiec M, Modrau IS, Dreifaldt M, Ericsson A, Holmgren A (2020). No-touch saphenous vein grafts in coronary artery surgery (SWEDEGRAFT): Rationale and design of a multicenter, prospective, registry-based randomized clinical trial. *American Heart Journal*.

[5] Taggart DP, Webb CM, Desouza A, Yadav R, Channon KM, De Robertis F (2018). Long-term performance of an external stent for saphenous vein grafts: the VEST IV trial. *Journal of Cardiothoracic Surgery*.

[6] Winkler B, Reineke D, Heinisch PP, Schönhoff F, Huber C, Kadner A (2016). Graft preservation solutions in cardiovascular surgery. *Interactive CardioVascular and Thoracic Surgery*.

[7] Pachuk CJ, Rushton-Smith SK, Emmert MY (2019). Intraoperative storage of saphenous vein grafts in coronary artery bypass grafting. *Expert Review of Medical Devices*.

[8] Harskamp RE, Alexander JH, Schulte PJ, Brophy CM, Mack MJ, Peterson ED (2014). Vein graft preservation solutions, patency, and outcomes after coronary artery bypass graft surgery: follow-up from the PREVENT IV randomized clinical trial. *JAMA Surgery*.

[9] Tsakok M, Montgomery-Taylor S, Tsakok T (2012). Storage of saphenous vein grafts prior to coronary artery bypass grafting: is autologous whole blood more effective than saline in preserving graft function. *Interactive CardioVascular and Thoracic Surgery*.

[10] Cartier R, Bouchard D, Latulippe JF, Buluran J (1995). Effect of solutions of preservation on the vascular reactivity of human saphenous veins. *Annales de Chirurgie*.

[11] Osmanagic-Myers S, Kiss A, Manakanatas C, Hamza O, Sedlmayer F, Szabo PL (2018). Endothelial progerin expression causes cardiovascular pathology through an impaired mechanoresponse. *Journal of Clinical Investigation*.

[12] Szabó PL, Dostal C, Pilz PM, Hamza O, Acar E, Watzinger S (2021). Remote Ischemic Perconditioning Ameliorates Myocardial Ischemia and Reperfusion-Induced Coronary Endothelial Dysfunction and Aortic Stiffness in Rats. *Journal of Cardiovascular Pharmacology and Therapeutics*.

[13] Clark JD, Gebhart GF, Gonder JC, Keeling ME, Kohn DF (1997). Special Report: The 1996 Guide for the Care and Use of Laboratory Animals. *ILAR Journal*.

[14] Aschacher T, Baranyi U, Aschacher O, Eichmair E, Messner B, Zimpfer D (2021). A Novel Endothelial Damage Inhibitor Reduces Oxidative Stress and Improves Cellular Integrity in Radial Artery Grafts for Coronary Artery Bypass. *Frontiers in Cardiovascular Medicine*.

[15] Veres G, Hegedűs P, Barnucz E, Zöller R, Klein S, Radovits T (2015). Graft preservation with heparinized blood/saline solution induces severe graft dysfunction. *Interactive CardioVascular and Thoracic Surgery*.

[16] O’Connell TX, Sanchez M, Mowbray JF, Fonkalsrud EW (1974). Effects on arterial intima of saline infusions. *Journal of Surgical Research*.

[17] Haime M, McLean RR, Kurgansky KE, Emmert MY, Kosik N, Nelson C (2018). Relationship between intra-operative vein graft treatment with DuraGraft® or saline and clinical outcomes after coronary artery bypass grafting. *Expert Review of Cardiovascular Therapy*.

[18] Perrault LP, Carrier M, Voisine P, Olsen PS, Noiseux N, Jeanmart H (2021). Sequential multidetector computed tomography assessments after venous graft treatment solution in coronary artery bypass grafting. *The Journal of Thoracic and Cardiovascular Surgery*.

[19] Caliskan E, de Souza DR, Böning A, Liakopoulos OJ, Choi Y, Pepper J (2020). Saphenous vein grafts in contemporary coronary artery bypass graft surgery. *Nature Reviews Cardiology*.

[20] Tekin I, Demir M, Özdem S (2022). Effect of different storage solutions on oxidative stress in human saphenous vein grafts. *Journal of Cardiothoracic Surgery*.

[21] Korkmaz-Icöz S, Ballikaya B, Soethoff J, Kraft P, Sayour AA, Radovits T (2021). Graft Preservation Solution DuraGraft® Alleviates Vascular Dysfunction Following In Vitro Ischemia/Reperfusion Injury in Rats. *Pharmaceuticals*.

[22] Gaudino M, Antoniades C, Benedetto U, Deb S, Di Franco A, Di Giammarco G (2017). Mechanisms, Consequences, and Prevention of Coronary Graft Failure. *Circulation*.

[23] Ben Ali W, Bouhout I, Perrault LP (2018). The effect of storage solutions, gene therapy, and antiproliferative agents on endothelial function and saphenous vein graft patency. *Journal of Cardiac Surgery*.

[24] Csanyi G, Bauer M, Dietl W, Lomnicka M, Stepuro T, Podesser BK (2006). Functional alterations in NO, PGI2 and EDHF pathways in the aortic endothelium after myocardial infarction in rats. *European Journal of Heart Failure*.

[25] Woodward LC, Antoniades C, Taggart DP (2016). Intraoperative Vein Graft Preservation: What Is the Solution. *The Annals of Thoracic Surgery*.

[26] Wilbring M, Tugtekin SM, Zatschler B, Ebner A, Reichenspurner H, Matschke K (2011). Even short-time storage in physiological saline solution impairs endothelial vascular function of saphenous vein grafts. *European Journal of Cardio-Thoracic Surgery*.

[27] Plass CA, Podesser BK, Prusa AM (2010). Effect of blower-mister devices on vasoreactivity of coronary artery bypass grafts. *The Journal of Thoracic and Cardiovascular Surgery*.

